# Assessment of the Protective Role of Prenatal Zinc versus Insulin Supplementation on Fetal Cardiac Damage Induced by Maternal Diabetes in Rat Using Caspase-3 and KI67 Immunohistochemical Stains

**DOI:** 10.1155/2016/7469549

**Published:** 2016-01-27

**Authors:** Ahmed S. Shams, Mona H. Mohammed, Mona M. Loka, Gamal M. Abdel Rahman

**Affiliations:** Department of Human Anatomy and Embryology, Faculty of Medicine, Suez Canal University, Ismailia 41111, Egypt

## Abstract

Maternal diabetes mellitus (DM) affects early organogenesis. Metabolic disorders of DM are associated with a depleted zinc status. This study evaluated the effect of maternal DM on cardiac development of rat fetuses and protective roles of prenatal zinc versus insulin supplementation. Pregnant rats were divided into 4 groups ((I) control, (II) STZ-induced DM, (III) STZ-induced DM treated with Zn, and (IV) STZ induced DM treated with insulin), all sacrificed on GD 20. Fetal heart weight of diabetic rats showed significant decrease compared to controls (*P* < 0.05). H&E stained section of controls had normal appearance of the myocardium, compared to diabetics that showed myocardial disarray with characteristic degenerative changes. Sections of zinc treated group showed restored architecture of normal myofibrils with minimal degenerative changes, while those of insulin treated group show partial restoration of the normal architecture of cardiomyocytes with focal improvement of cardiac tissue. Caspase-3 immunostained slides showed positive cytoplasmic immunoreactivity in diabetic group. But KI67 immunostained slides revealed negative nuclear immunoreaction in diabetics. We observed that gestational diabetes was associated with increased risk of fetal myocardial damage that might be caused by increased apoptotic level. Treating diabetic pregnant subjects with zinc and insulin was associated with improvement in myocardial integrity.

## 1. Introduction

Global prevalence of DM in 2010 among adults has been reported to be 6.4% [1]. Maternal diabetes affects early organogenesis, leading to congenital malformations in various organs including the heart [2]. Moreover, it leads to massive cell damage, increase in apoptosis, and increased risk to develop cardiovascular malformation in the offspring [3, 4].

Several studies considered hyperglycemia as an independent risk factor directly causing cardiac damage, leading to diabetic cardiomyopathy [5, 6]. Hyperglycemia causes cellular oxidative damage by increasing production of ketone bodies, which lead to generation of reactive oxygen species (ROS). Oxidative stress occurs as a result of an imbalance between the production of ROS and their neutralization by antioxidants; the increased level of ROS causes the free radical generation and decreased antioxidants or both, which influence organogenesis during development [7]. In addition, ROS cause activation of caspase-3 [8].

Alterations of antioxidant micronutrient status have been reported in subjects with type 1 or 2 diabetes mellitus [9]. Metabolic disorders of diabetes are associated with a depleted zinc status that increases the susceptibility of the embryonic heart to oxidative damage [10]. Correction of zinc deficiency in subjects with type 1 DM leads to decreased lipid peroxidation and improvements in glucose homeostasis [11]. Furthermore, impairments of zinc status have been reported as risk factors in the progression of diabetes and its complication [12].

Therefore, this study was conducted to assess the effect of maternal diabetes on the cardiac development of albino rat fetuses and the roles of prenatal zinc/or insulin administration in counteracting this effect.

## 2. Materials and Methods 

### 2.1. Type of Study and Grouping of Rats

Experimental study included four groups (6/group) of female albino rats. Group (I) was the control group and groups (II), (III), and (IV) were the diabetic groups. Thirty-eight fetuses were collected from the pregnant mothers of each group on Gestational Day (GD) 20.

Diabetes was induced in the virgin female albino rats of groups (II) to (IV) by a single dose of intraperitoneal administration of streptozotocin (40 mg/kg). Blood glucose was measured 4 days after STZ injection from the tail vein using ultra one touch device and rats with blood glucose levels exceeding 200 mg/dL were considered diabetic.

After induction of diabetes, mating was carried out by placing two female rats with one male rat in a cage overnight. Pregnancy was verified by the presence of spermatozoa in the vaginal smears, taken from the mated females, which denotes the first day of gestation (GD1) and the assignment of groups were as follows.

*Group (I) (Control Group)*. This group included nondiabetic pregnant rats with blood glucose level less than 200 mg/dL.

*Group (II) (STZ-Induced DM Group)*. This group included pregnant rats with glucose level more than 200 mg/dL throughout pregnancy till GD 20.

*Group (III) (STZ-Induced DM Treated with Zn Group)*. This group included pregnant rats that received daily Zn sulfate (5 mg/kg body weight) diluted in normal saline intraperitoneally from GD 1 to GD 15.

*Group (IV) (STZ-Induced DM Treated with Insulin Group)*. This group included pregnant diabetic rats that received a daily dose of 15 IU/kg mixtard insulin subcutaneously till GD 20.

In all cases, the pregnant rats were sacrificed by an overdose of ether on GD 20 and the fetuses were obtained through caesarian section.

The fetuses were dissected out; the viable fetuses and placentas were weighed. Fetal hearts were dissected out, weighed, collected, and fixed in 4% paraformaldehyde in the phosphate buffered saline for 1~6 hours. Then, the fixed hearts were embedded by paraffin and sectioned following routine practice. The sections were manipulated to be 5 *μ*m in thickness for following use.

#### 2.1.1. Hematoxylin and Eosin (H&E) Staining

H&E staining was performed using routine method. Sections were examined by light microscope to comment on the general architecture of the myocardial tissue.

#### 2.1.2. Immunohistochemical Staining

Paraffin embedded sections were also analyzed by caspase-3 immunostain (present in apoptotic cells) and polyclonal anti-ki 67 antibody (present only in cycling cells to estimate the degree of cardiomyocyte proliferation).

#### 2.1.3. Morphometric Study

*(i) H&E Morphometric Analysis*. H&E qualitative analysis determined by direct visual counting of vacuolar degenerative changes in ten fields (mean values) for each of three slides per sample at 40x magnification and the following grades were used [13]:

- No change: normal cardiac microscopy.
- Mild change: vacuolar degeneration in 5–15% of cells.
- Moderate change: vacuolar degeneration in 16–35% of cells.
- Marked change: vacuolar degeneration in more than 35% of cells.

*(ii) Immunohistochemical Morphometric Analysis*. The apoptotic cells and bodies (caspase-3 immunohistochemically positive cells) were counted in five high-power fields (400x magnification). The apoptotic index (AI) was calculated as the percentage of positively stained cells using the following equation: AI is number of apoptotic cells/total number of nucleated cells.

The apoptotic index was determined as follows [14, 15]:

- Grade I: apoptotic cells not detected or AI < 1%.
- Grade II: AI < 5%.
- Grade III: AI: 5–10%.
- Grade IV: AI > 10%.

The proliferating cells (KI67 immunohistochemically positive cells) were counted in five high-power fields (400x magnification) for each section; then, proliferation index was calculated by the percentage of positive cells in each field as follows [16]:

- Low proliferation KI67 index ≤ 15%.
- Intermediate proliferation KI67 index 16–30%.
- High proliferation KI67 index > 30%.

*Ethical Considerations*. All animal experiments were performed according to the guidelines and approval of the local Ethics Animal Review Board.

### 2.2. Statistical Analysis

The data were analyzed using SPSS version 16.0. Quantitative parametric variables were presented by mean and standard deviation (SD) and compared by Student's *t*-test (comparing means of control group to means of other groups) and ANOVA test (comparing means of more than two groups, with Dunnett's multiple comparison as a post hoc test). Qualitative variables were compared by the Chi-square test. A probability level of *P* < 0.05 was designated as significant in this study.

## 3. Results

### 3.1. Assessment of the Maternal and Fetal Random Blood Glucose Levels in Different Groups

A statistically significant increase in both maternal and fetal random blood glucose was observed in STZ-induced DM group when compared to the control, STZ-induced DM treated with zinc, and STZ-induced DM treated with insulin groups (Table 1).

Moreover, the maternal random blood glucose showed a statistically significant increase in STZ-induced DM treated with zinc group when compared to the control one and STZ-induced DM treated with insulin group (Table 1).

### 3.2. Assessment of Pregnancy Outcome and Embryolethality in the Different Groups

Table 2 showed that STZ-induced DM group had a statistically significant increase in the mortality rate compared to control, STZ-induced DM treated with zinc, and STZ-induced DM treated with insulin groups. STZ-induced DM group had a statistically significant increase in the total number of corpora lutea compared to control group. Moreover, resorption was found in a statistically significant higher level in the STZ-induced DM group compared to the control group. Administration of both zinc and insulin resulted in improvement of the mortality rate, number of corpora lutea, and the rate of resorption. However, this improvement did not reach that of the control group.

The differences in these parameters (number of corpora lutea and rate of resorption) between STZ-induced DM group and STZ-induced DM treated with both zinc and insulin groups were proved to be insignificant (Table 2).

Regarding number of dead fetuses, a statistically significant increase was found in STZ-induced DM group when compared to the control group, while a statistically nonsignificant difference was observed between STZ-induced DM group and both STZ-induced DM treated with zinc and STZ-induced DM treated with insulin groups (Table 2).

### 3.3. Assessment of the Maternal Status at Termination of Pregnancy in the Different Groups

*(i) Maternal Weight*. The maternal final body weight showed a statistically significant decrease in the STZ-induced DM group when compared to the control, STZ-induced DM treated with zinc, and STZ-induced DM treated with insulin groups. Additionally, the final body weight had statistically significant lower levels in both STZ-induced DM treated with zinc and STZ-induced DM treated with insulin groups when compared to the control group (Table 3), with a statistically nonsignificant difference on comparing both STZ-induced DM treated with zinc and STZ-induced DM treated with insulin groups (Table 3).

Regarding maternal weight gain, a statistically significant increase was proved in the control group compared to the STZ-induced DM, STZ-induced DM treated with zinc, and STZ-induced DM treated with insulin groups. On the contrary, a statistically significant decrease was found in the maternal weight gain in STZ-induced DM group when compared to STZ-induced DM treated with zinc and the STZ-induced DM treated with insulin groups (Table 3).

### 3.4. Assessment of the Fetal Growth in the Different Groups

*(i) Placental Weight*. Placental weight had a statistically significant increase in the STZ-induced DM group compared to both STZ-induced DM treated with zinc and STZ-induced DM treated with insulin groups (Table 3).

*(ii) Fetal Weight*. Fetal weight showed a statistically significant decrease in STZ-induced DM group compared to the control, STZ-induced DM treated with zinc, and STZ-induced DM treated with insulin groups (Table 3).

*(iii) Fetal Heart Weight*. Fetal heart weight showed a statistically significant decrease in STZ-induced DM group compared to the control, STZ-induced DM treated with zinc, and STZ-induced DM treated with insulin groups (Table 3).

A statistically nonsignificant difference was found on comparing fetal heart weight of the control group with both the STZ-induced DM treated with zinc and the STZ-induced DM treated with insulin groups (Table 3).

Moreover, on comparing the fetal heart weight of the STZ-induced DM treated with zinc group with that of the STZ-induced DM treated with insulin group, a statistically nonsignificant difference was evident (Table 3).

*(i) Histological and Immunohistochemical Results*

**Figure 1:**
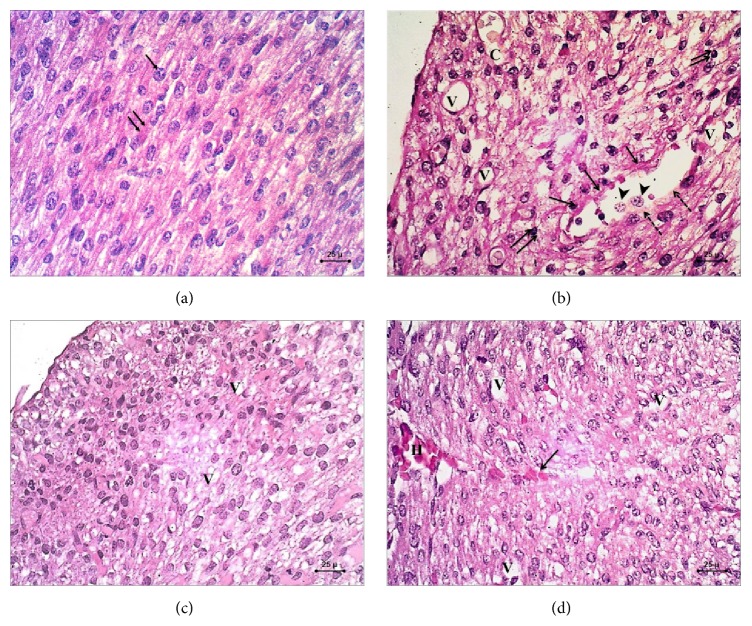
Photomicrograph of H&E (×400) sections in the fetal myocardium. (a) Control group showing normal striated pattern of the cardiomyocytes with centrally located nuclei (arrow) and eosinophilic cytoplasm (double arrow). (b) STZ-induced DM group showing myocardial disarray, marked vacuolar degeneration of the cardiomyocytes (V), fragmentation of the nuclei of cardiomyocytes (arrow) and pyknotic changes of the nuclei (arrow head), congested blood vessels (C), and discontinuation of the vessel wall (dashed arrow) with the presence of subendocardial thickening (double arrow). (c) STZ-induced DM treated with zinc group showing restored architecture of cardiomyocytes with the presence of mild vacuolar degeneration (V). (d) STZ-induced DM treated with insulin group showing focal improvement of the myocardium with the presence of vacuolar degeneration (V) and myocardial hemorrhage (H) with extravasated red blood cells within the myocardial tissue (arrow).

*(a) The Control Group*. H&E stained sections of the control group showed the normal appearance of the myocardial tissue having branching cardiac muscle cells with centrally located nuclei and pale staining cytoplasm. There are darker transverse lines found at irregular interval between the cardiac muscle cells. These are the intercalated discs representing the interface between adjacent cardiac muscle cells (Figure 1(a)).

**Figure 2:**
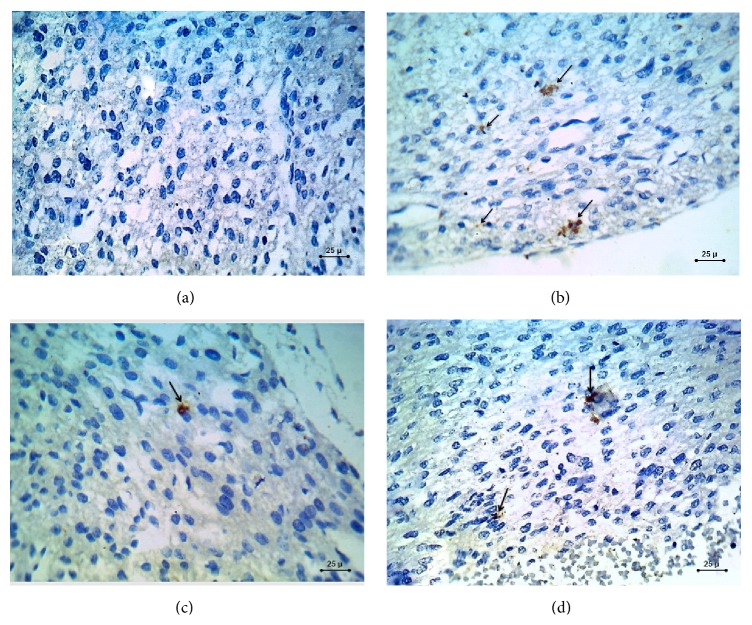
Photomicrograph of caspase-3 immunohistochemical staining (×400) sections in the fetal myocardium. (a) control group. (b) STZ-induced DM group. (c) STZ-induced DM treated with zinc group. (d) STZ-induced DM treated with insulin group. Shown is positive brownish cytoplasmic immunohistochemical staining with caspase-3 in group (b) which seems to be downregulated in both group (c) and group (d); this brownish cytoplasmic immunohistochemical staining is almost absent in group (a).

**Figure 3:**
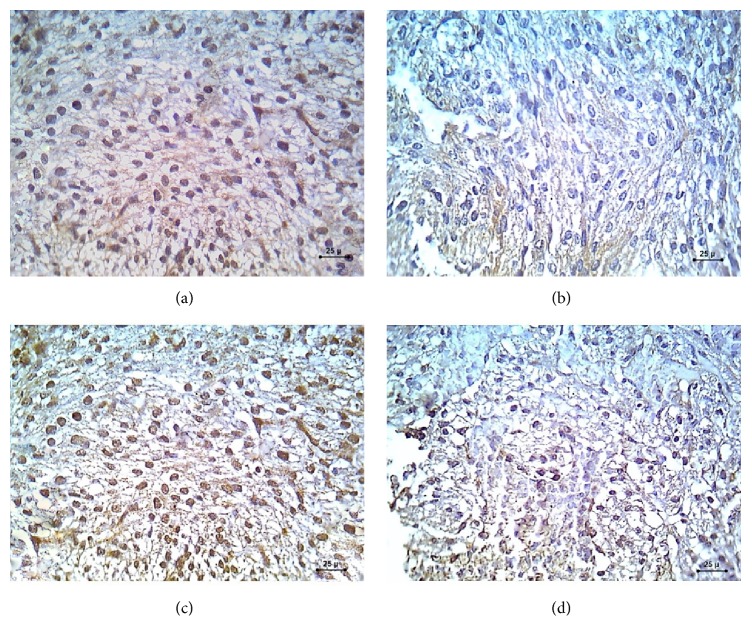
A photomicrograph of KI67 immunohistochemical staining (×400) sections in the fetal myocardium. (a) Control group showing dense positive brown nuclear immunohistochemical staining with KI67. (b) STZ-induced DM group showing negative nuclear immunohistochemical staining with KI67. (c) STZ-induced DM treated with zinc group showing moderate positive nuclear immunohistochemical staining with KI67. (d) STZ-induced DM treated with insulin group showing minimal positive nuclear immunohistochemical staining with KI67.

Myocardial sections of the control group stained with caspase-3 immunostain showed negative brown cytoplasmic immunoreaction (Figure 2(a)), while KI67 stained sections showed dense positive brown nuclear immunoreaction (Figure 3(a)).

*(b) The STZ-Induced DM*. H&E stained sections of the STZ-induced diabetic group showed myocardial disarray accompanied with loss of the normal striated architecture of the cardiomyocytes with presence of characteristic degenerative changes of the nuclei in the form of pyknosis, karyorrhexis, and karyolysis (Figure 1(b)).

The myocardial sections stained with caspase-3 immunostain showed positive brown cytoplasmic caspase-3 immunoreactivity (Figure 2(b)), while sections stained with KI67 immunostain showed negative brown nuclear immunoreaction (Figure 3(b)).

*(c) The STZ-Induced DM Treated with Zinc Group*. H&E stained sections of the STZ-induced DM treated with zinc group showed restored architecture of normal myofibrils and orientation of myocytes. Minimal degenerative changes with moderate vacuolar degeneration were still evident (Figure 1(c)).

Very few focal areas of hemorrhage and vascular congestion were also seen (Figure 1(c)).

Myocardial sections stained with caspase-3 immunostain showed minimal positive brown cytoplasmic caspase-3 immunoreactivity (Figure 2(c)), while the myocardial sections stained with KI67 immunostain showed moderate positive brown nuclear immunoreaction (Figure 3(c)).

*(d) The STZ-Induced DM Treated with Insulin Group*. H&E stained sections of the STZ-induced DM treated with insulin group show partial restoration of the normal architecture of cardiomyocytes with focal improvement of the cardiac tissue. Marked vascular congestion accompanied with hemorrhage and multiple vacuolar degenerations were evident (Figure 1(d)).

Myocardial sections stained with caspase-3 immunostain showed moderate positive brown cytoplasmic caspase-3 immunoreactivity (Figure 2(d)), while sections stained with KI67 immunostain showed minimal positive brown nuclear immunoreaction (Figure 3(d)).

*(ii) Morphometric Results*

*(a) Frequency Distribution of Vacuolar Degenerative Changes in the Different Groups*. Very few areas (5%) of H&E stained sections in the control group showed mild vacuolar degenerative changes, while 80% of sections in STZ-induced DM group had marked vacuolar degenerative changes. Only 5% of sections in STZ-induced DM treated with zinc group had marked vacuolar degenerative changes and about 18% in STZ-induced DM treated with insulin group had marked vacuolar degenerative changes (Table 4).

*(b) Percentage Frequency Distribution of Apoptotic Index (AI) in Caspase-3 Immunohistochemical Stained Myocardial Sections in the Different Groups*. A statistically significant increase was observed in STZ-induced DM group compared to the control, STZ-induced DM treated with zinc, and STZ-induced DM treated with insulin groups (Table 5).

*(c) Percentage Frequency Distribution of Proliferation Index in KI67 Immunohistochemical Stained Myocardial Sections in the Different Groups*. A statistically significant decrease was observed in STZ-induced DM group compared to the control, STZ-induced DM treated with zinc, and STZ-induced DM treated with insulin groups. A statistically significant increase was noted on comparing the control group to STZ-induced DM treated with zinc and STZ-induced DM treated with insulin groups (Table 6).

## 4. Discussion

In the current study, the maternal blood glucose levels of diabetic (360.00 mg/dL) and Zn-supplemented diabetic (180.00 mg/dL) groups were significantly higher than those of the control (108.33 mg/dL) and insulin treated (115.83 mg/dL) groups. Moreover, the fetal blood glucose level of diabetic (334.67 mg/dL) group was significantly higher than that of the control (96.67 mg/dL), Zn-supplemented diabetic (123.17 mg/dL), and insulin treated (91.67 mg/dL) groups. We observed that administration of zinc during pregnancy improved both the maternal and fetal random blood sugar but did not reach the levels of the control and insulin treated groups. In parallel to our study, the study of Kumar et al. in Singapore reported that maternal blood glucose levels of diabetic and zinc-supplemented diabetic groups were higher than those of the control group [17].

The present study revealed that STZ-induced DM group showed a statistically significant increase in the mortality rate compared to the control, STZ-induced DM treated with zinc, and STZ-induced DM treated with insulin groups. Additionally, resorption rate was found in significantly higher level in the STZ-induced DM group compared to the control group. Administration of both zinc and insulin resulted in improvement of the mortality rate and the rate of resorption. However, this improvement did not reach that of the control group.

Regarding the number of dead fetuses, a significantly higher level was noted in STZ-induced DM group compared with the control group, with statistically nonsignificant difference observed compared with STZ-induced DM treated with zinc group and STZ-induced DM treated with insulin group. These data were supported by several studies [18, 19]. More recent study seems also to be in concordance with the current study which is that of Kumar et al. which demonstrated reduced number of embryos observed in diabetic pregnancies compared to control and Zn-supplemented diabetic groups [17].

The reduction in number of embryos observed in diabetic pregnancies could be due to either reduced fecundity or early embryonic lethality resulting from a higher malformation rate in the embryos of diabetic mothers compared with those of nondiabetic mothers as explained by Phelan et al. [18]. The increased number of embryos from zinc-supplemented diabetic group may possibly explain that zinc supplement reduces early embryonic lethality resulting from a higher mortality rate in the embryos of diabetic mothers compared with those of zinc-supplemented diabetic mothers [17]. Furthermore, Kumar et al. reported that superoxide anions were significantly increased in the diabetes group whereas they were decreased in the zinc-supplemented diabetic group. They added that alteration may be because zinc supplementation significantly decreased apoptosis and the levels of reactive oxygen species (ROS) in addition to the antioxidant activity of zinc [17, 20].

Zinc is a transition metal which may protect against oxidation of vital compounds and inhibit production of reactive oxygen species [21]. Several studies have shown that the levels of zinc are lower in women with gestational diabetes mellitus [22].

Additionally, embryonic lethality rate was found to be decreased by the administration of insulin during pregnancy which corrects the hyperglycemia, a result that seems concordant with the study of [23].

In the current study, there was no weight difference between any of the experimental groups on the day of conception. In general, all groups gained weight during pregnancy. The control group, STZ-induced DM treated with zinc group, and STZ-induced DM treated with insulin group showed greater increase in maternal body weight than the STZ-induced DM group. The increase in body weight in STZ-induced DM treated with zinc group was less than the control group; these results coincide with the studies of Kumar et al. which observed that all studied groups gained weight during pregnancy and the STZ-induced DM treated with zinc group was higher than the diabetic group [17, 24].

The present study also showed that fetal body weight had significantly lower value in the fetuses of diabetic mothers compared with fetuses of control, STZ-induced DM treated with zinc, and STZ-induced DM treated with insulin groups. This agrees with the studies of Simán et al. [10, 24]. On the contrary, Van Assche et al. found that, in rats as in humans, increased fetal weight in diabetics was noticed at the end of gestation [25–27].

Some of the reduced fetal weight observed in the present study possibly occurs due to the production of reactive oxidative species, which was preventable with antioxidant treatment, whereas the remaining growth retardation may have resulted from other non-ROS-related mechanisms as the availability of nutrients and oxygen to the fetus, intrauterine insults, and a variety of growth factor and proteins of maternal, fetal, and placental origin most of which are disturbed with diabetic pregnancy as explained by Kumar et al. [17].

By comparing the fetal body weight in the zinc and insulin treated groups with the control group, we found insignificant difference indicating the protective role of zinc and insulin in the diabetic embryopathy which coincides with the results of He et al. [10, 20].

Regarding the placental weight, it showed greater values in the diabetic group when compared with the control, zinc treated, and insulin treated groups. This result is in match with the results of several studies as Eriksson et al. [23, 28]. Other investigators clarified that placentas from rats with manifested diabetes were heavier than the placentas from normal rats which might represent a compensatory mechanism to assure the maternal-fetal exchanges contributing to fetal development [28].

In addition, the current study revealed that the fetal and placental weights of the insulin treated diabetic group were nearly similar to those of the control group and this was in concordance with the study of Ericsson et al. [26].

On examination of the Hematoxylin and Eosin stained sections of the diabetic group, it revealed myocardial disarray accompanied with loss of the normal striated architecture of the cardiomyocytes with presence of characteristic degenerative changes of the nuclei in the form of pyknosis, karyorrhexis, and karyolysis. These results coincide with the results of Schneider et al. [29]. On the contrary, Russell et al. reported hypertrophy and hyperplasia of the cardiomyocytes and fibroblasts in the fetal hearts from diabetic pregnancy experience [30, 31].

Moreover, most of the fetal myocardium of the diabetic group showed marked vacuolar degeneration of the cardiomyocytes, accompanied with marked vascular congestion, hemorrhage, subendocardial thickening, and vascular wall thickening. These results coincide with that of the Mexican study done by Manjarrez et al. which reported the presence of dilated sinusoids with endothelial lining in the myocardium, after application of STZ [32].

The insulin treated diabetic group showed only partial improvement but there still exist moderate vacuolar degenerative changes of the myocytes, congestion, and hemorrhage. Regarding zinc treated diabetic group, the fetal myocardium showed marked improvement in the myocytes, minimal degenerative changes, and restored architecture of normal myofibrils. The previous findings seem to be concordant with the study of Kumar et al. who found that the structure of the myocardium was better preserved in the hearts of diabetic rats treated with zinc. They reported reduced numbers and spread of cardiomyocytes degeneration, which mostly showed normal nuclei and homogeneous cytoplasm. The frequency of fibrotic scars and accumulation of interstitial and perivascular connective tissue were clearly diminished in the zinc-protected group. Moreover, there were restored normal myofibril architecture and orientation of myocytes with decreased cardiac anomalies [17].

The precise mechanism by which zinc antagonizes the cardiac teratogenic effects of diabetes remains uncertain. However, it is likely to be the result of an effect on several metabolic functions that are associated with antioxidation. Zinc protects cells from abnormal synthesis of nucleic acids and proteins, chromosomal defects, and impairment of cellular growth and morphogenesis [33]. Zinc induces the production of antioxidative substances, such as metallothionein [34]. Moreover, zinc reduces peroxidation of unsaturated fatty acids, prevents excessive lipid peroxidation of cellular membranes, and stabilizes the normal construction and function of cellular membrane systems by inhibiting free radicals [35]. All these mechanisms may help to explain the antioxidative effects of zinc that were observed in the current study.

Immunohistochemical staining with caspase-3 immunostain, which is an important indicator of apoptotic cell death, showed positive brown cytoplasmic caspase-3 immunoreactivity in the fetal heart of diabetic mothers as compared with control group (negative brown cytoplasmic immunoreaction). Minimal positive brown cytoplasmic caspase-3 immunoreactivity and moderate positive brown cytoplasmic caspase-3 immunoreactivity were observed in the zinc treated diabetic group and insulin treated diabetic groups, respectively. These results suggest that apoptosis via caspase-3 activation, which occurred in the myocardium of diabetic fetuses, can be minimized by administration of therapeutic doses of zinc and insulin during pregnancy. This is supported by the results of the studies done by Kumar et al. [17, 36].

Apoptosis of cardiac muscle cells and endothelial cells has been observed in the heart of patients with diabetes [37] and in STZ-induced diabetic rats [38] and mice [39].

On the other hand, immunohistochemical staining of the fetal heart sections with KI67 was done to assess the cellular proliferation. The current results revealed negative brown nuclear immunoreaction indicating minimal proliferative cells in the fetuses of the diabetic mothers compared to dense positive brown nuclear immunoreaction, moderate positive brown nuclear immunoreaction, and minimal positive brown nuclear immunoreaction in the control, zinc treated diabetic, and insulin treated diabetic groups, respectively. These results seem concordant with the results of Song et al. [40]. Additionally, Song et al. stated that insulin administration decreases the number of apoptotic cells and increases the number of proliferative cells [40]. However, Miao et al. found an increase in the apoptotic cell death, reflected by terminal deoxynucleotidyl transferase dUTP nick end labeling (TUNEL) positive cells and cell proliferation, reflected by Ki-67 positive nuclear in the aorta of diabetic mice, but not in zinc treated diabetic mice [41].

The present study revealed that embryonic cardiac tissues respond differently to the adverse environment created by maternal diabetes during pregnancy. We found that treating hyperglycemia by insulin ameliorates myocardial morphological abnormalities and partially inhibits myocardial cell death. Our study also suggests that Zn has antiapoptotic activity against glucose-induced myocardial disarrangement caused by decreased cellular proliferation and increased apoptotic level. The adverse effects of maternal diabetes on an unborn fetus could possibly be treated by insulin and Zn supplementation in the early stages of gestational diabetic pregnancy.

## 5. Conclusion

Gestational diabetes was associated with increased risk of fetal myocardial damage that might be caused by decreased cellular proliferation and increased apoptotic level. Treating diabetic pregnant females with both therapeutic doses of zinc and insulin was associated with improvement in myocardial integrity. However, STZ-induced DM treated with zinc group showed better improvement than STZ-induced DM treated with insulin group.

## Figures and Tables

**Table 1:** Mean ± SD of maternal and fetal random blood glucose levels in the different groups.

Parameter	Group				ANOVA	
	C	STZ-DM	STZ-DM + Zn	STZ-DM + In	*F*	*P* value
Maternal random blood glucose (mg/dL)	108.33 ± 11.25	360.00 ± 63.48^*∗*a^	180.00 ± 38.34^*∗*a,b^	115.83 ± 32.00^*∗*b,c^	49.5	<0.00001

Fetal random blood glucose (mg/dL)	96. 67 ± 7.53	334. 67 ± 63.19^*∗*a^	123.17 ± 40.31^*∗*b^	91.67 ± 17.52^*∗*b^	54.2	<0.00001

C: control group, STZ-DM: streptozotocin-induced diabetic group, STZ-DM + In: streptozotocin-induced diabetic treated with insulin group, and STZ-DM + Zn: streptozotocin-induced diabetic treated with Zn group.

^*∗*^ *P* < 0.05, ^a^compared to the control group, ^b^compared to the STZ-DM group, and ^c^compared to the STZ-DM + Zn group.

**Table 2:** Pregnancy outcome and embryolethality in the different groups.

Parameter	Group								Chi-square	*P* value
	C		STZ-DM		STZ-DM + Zn		STZ-DM + In			
	No.	%	No.	%	No.	%	No.	%		
Total rats	6	100	12	100	8	100	8	100	—	—

Mortality rate	0	0	6^*∗*a^	50	2^*∗*b^	25	2	25^*∗*b^	66.67	<0.00001

Total number of corpora lutea (in metrial gland)	39		50^*∗*a^		42		42		2.99	0.56

Resorptions	1	2.6	12^*∗*a^	24	4	9.5	2	4.8	28.62	<0.00001
Total number of fetuses	38	97.4	38	76	38	90.5	40	95.2		

Live fetuses	38	100	31	81.6	36	94.7	36	90	23.35	0.000034
Dead fetuses	0	0	7^*∗*a^	18.4	2	5.3	4	10		

C: control group, STZ-DM: streptozotocin-induced diabetic group, STZ-DM + In: streptozotocin-induced diabetic treated with insulin group, and STZ-DM + Zn: streptozotocin-induced diabetic treated with Zn group.

^*∗*^ *P* < 0.05, ^a^compared to the control group and ^b^compared to the STZ-DM group.

**Table 3:** Mean ± SD of the maternal, placental, fetal, and fetal heart weights in the different groups.

Parameter	Group				ANOVA	
	C	STZ-DM	STZ-DM + Zn	STZ-DM + In	*F*	*P* value
Maternal weight gain (gm)	47.5 ± 3.08	33.83 ± 2.93^*∗*a^	41.33 ± 3.98^*∗*a,b^	38.17 ± 2.79^*∗*a,b^	19.04	<0.00001
Placental weight (gm)	0.565 ± 0.081	0.624 ± 0.211	0.545 ± 0.092^*∗*b^	0.540 ± 0.087^*∗*b^	3.39	0.020
Fetal weight (gm)	3.29 ± 0.21	2.98 ± 0.33^*∗*a^	3.16 ± 0.44	3.21 ± 0.42^*∗*b^	4.89	0.003
Fetal heart weights (gm)	0.479 ± 0.062	0.424 ± 0.071^*∗*a^	0.461 ± 0.095	0.475 ± 0.075^*∗*b^	4.34	0.006

C: control group, STZ-DM: streptozotocin-induced diabetic group, STZ-DM + In: streptozotocin-induced diabetic treated with insulin group, and STZ-DM + Zn: streptozotocin-induced diabetic treated with Zn group.

^*∗*^ *P* < 0.05, ^a^compared to the control group and ^b^compared to the STZ-DM group.

**Table 4:** Percentage of frequency distribution of vacuolar degenerative changes in the different groups.

Parameter	Group			
	C	STZ-DM	STZ-DM + Zn	STZ-DM + In
No change (%)	95	0	2	2
Mild change (%)	5	5	80	25
Moderate change (%)	0	15	13	55
Marked change (%)	0	80	5	18
Total (%)	100	100^*∗*a^	100^*∗*b^	100^*∗*a,b^

Chi-square 637.9.

*P* value < 0.00001.

C: control group, STZ-DM: streptozotocin-induced diabetic group, STZ-DM + In: streptozotocin-induced diabetic treated with insulin group, and STZ-DM + Zn: streptozotocin-induced diabetic treated with Zn group.

^*∗*^ *P* < 0.05, ^a^compared to the control group and ^b^compared to the STZ-DM group.

**Table 5:** Percentage frequency distribution of apoptotic index (AI) in caspase-3 immunohistochemical stained myocardial sections in the different groups.

Parameter	Group			
	C	STZ-DM	STZ-DM + Zn	STZ-DM + In
Grade I AI < 1%	88	0	8	5
Grade II AI < 5%	10	2	72	25
Grade III AI: 5–10%	2	23	15	55
Grade IV AI > 10%	0	75	5	15
Total (%)	100	100^*∗*a^	100^*∗*a,b^	100^*∗*a,b,c^

Chi-square 533.86.

*P* value < 0.00001.

C: control group, STZ-DM: streptozotocin-induced diabetic group, STZ-DM + In: streptozotocin-induced diabetic treated with insulin group, and STZ-DM + Zn: streptozotocin-induced diabetic treated with Zn group.

^*∗*^ *P* < 0.05, ^a^compared to the control group, ^b^compared to the STZ-DM group, and ^c^compared to the STZ-DM + Zn group.

**Table 6:** Percentage frequency distribution of proliferation index in KI67 immunohistochemical stained myocardial sections in the different groups.

Parameter	Group			
	C	STZ-DM	STZ-DM + Zn	STZ-DM + In
Low proliferation KI67 index ≤ 15%	12	68	23	27
Intermediate proliferation KI67 index 16–30%	25	17	47	46
High proliferation KI67 index >30%	63	15	30	27
Total (%)	100	100^*∗*a^	100^*∗*a,b^	100^*∗*a,b^

Chi-square 113.17.

*P* value < 0.00001.

C: control group, STZ-DM: streptozotocin-induced diabetic group, STZ-DM + In: streptozotocin-induced diabetic treated with insulin group, and STZ-DM + Zn: streptozotocin-induced diabetic treated with Zn group.

^*∗*^ *P* < 0.05, ^a^compared to the control group and ^b^compared to the STZ-DM group.
